# THE ROLE OF MEDITERRANEAN DIET IN THE PRIMARY AND SECONDARY PREVENTION OF ATRIAL FIBRILLATION: A SCOPING REVIEW

**DOI:** 10.1093/geroni/igac059.3051

**Published:** 2022-12-20

**Authors:** Yan Su, Kuan-Ching Wu, Oleg Zaslavsky, Marilyn Prasun

**Affiliations:** Illinois State University, Normal, Illinois, United States; University of Washington, Seattle, Washington, United States; Unversity of Washington, Seattle, Washington, United States; Illinois State University, Normal, Illinois, United States

## Abstract

Atrial fibrillation (AF) is the most common cardiac arrhythmia, especially in patients older than 65. The prevalence of AF increased 3-fold over the last 50 years. All treatments, such as cardioversion, ablation, and anticoagulation, are associated with risks and relapses. Besides, these strategies may not apply to all patients with AF. Adherence to the Mediterranean dietary pattern is assumed as an ideal nutritional model for AF. Yet, there is a gap on 1) how adherence to the Mediterranean diet prevents AF and 2) how the Mediterranean diet affects complications in patients with AF. A scoping review was performed in June 2022 to identify studies focusing on the Mediterranean diet and AF. A total of 334 articles were retrieved and 12 met inclusion criteria. Six studies were interventions (n=6) and six were observational studies (n=6). In the six intervention studies, five were nested within the PREDIMED (Prevención con Dieta Mediterránea) study. In AF occurrence (n=6), the results were inconsistent. The Mediterranean diet plus extra virgin olive oil was significantly and favorably related to AF, however, Mediterranean diet plus nuts was not, when compared with the low-fat diet; Mediterranean diet modified how long chain acylcarnitine affected development of AF but not how tryptophan or arginine metabolites affected AF. In patients diagnosed with AF (n=6), Mediterranean diet favorably related to adverse cardiovascular events, oxidative stress, and Mediterranean diet adherence, and did not affect anticoagulation for those taking anticoagulants. Further research is needed to examine how the Mediterranean diet affects the development of AF.

